# Total hip arthroplasty through the direct anterior approach with and without the use of a traction table: a matched-control, retrospective, single-surgeon study

**DOI:** 10.1186/s13018-020-02184-6

**Published:** 2021-01-11

**Authors:** Diane Wernly, Julien Wegrzyn, Geoffroi Lallemand, Jaad Mahlouly, Christophe Tissot, Alexander Antoniadis

**Affiliations:** Service of Orthopedic Surgery and Traumatology, Lausanne University Hospital and University of Lausanne, Avenue Pierre-Decker 4, 1010 Lausanne, Switzerland

**Keywords:** Total hip arthroplasty, Direct anterior approach, Traction table, Complications, Leg length

## Abstract

**Background:**

Hip surgeons performing total hip arthroplasty (THA) through the direct anterior approach (DAA) commonly use a traction table to facilitate exposure. Even though performing THA through DAA without a traction table could be technically more demanding, this technique offers the advantage of intraoperative leg length comparison. Therefore, this study aimed to compare clinical outcomes, complication rates, component positioning, and leg length discrepancy (LLD) after THA through the DAA performed with or without a traction table.

**Methods:**

A single-surgeon continuous series of 75 patients who underwent DAA THA performed with a traction table was matched for gender, age, and BMI with 75 patients who underwent DAA THA performed without a traction table (male, 62; female, 88, with an average age of 68 years old). Clinical and radiological outcomes, intra- and postoperative complications, and LLD were retrospectively assessed.

**Results:**

No statistically significant difference was detected in surgical time, hospital stay, Harris Hip Score (HHS), complication rates, and implant positioning between the two groups. Leg length restoration was significantly more accurate in the group performed without a traction table (2.4 ± 2 mm vs. 3.7 ± 3.1 mm; *p* value ≤ 0.05). No LLD > 10 mm was reported in the group performed without a traction table, whereas two cases (2.7%) were reported in those performed with a traction table.

**Conclusion:**

Performing THA through DAA without a traction table was associated with a significantly more accurate leg length restoration without a significant increase in the rates of intra- and postoperative complications.

## Background

Over the past years, several surgical approaches for THA have been described with the trend going towards minimally invasive surgery [1, 2]. Current practice analysis based on reviews from national joint registries demonstrates that most surgeons prefer the posterior or the direct lateral approach to the hip [3]. However, the direct anterior approach (DAA) is gaining popularity owing to its soft tissue-preserving nature, the low risk of dislocation, and the accurate placement of the components [4–8]. A recent meta-analysis shows that compared with the posterior approach, DAA presents superior early recovery following THA [9–11]. Although being first described by Hueter in 1882 [12, 13], the Judet brothers were the first to use a specially designed traction table to perform a THA through DAA in 1985 [14]. In the 2000s, the interest for the DAA in THA was renewed with Matta developing his own traction table [15, 16]. Currently, the DAA is the most common approach in Switzerland for THA (35%) with the majority of them being performed with the use of a traction table [7].

The traction table offers the advantages of reducing the number of required assistants or even performing the surgery without an assistant, but most importantly might facilitate the femoral exposure that could be challenging in the DAA particularly during the learning curve [2, 17, 18]. However, traction tables can also have some disadvantages such as exerting tremendous forces with several reports of trochanteric, femoral, and ankle fractures, as well as neurapraxia [10, 15, 19, 20]. Furthermore, traction tables are expensive, require additional logistics to be stored, might increase the surgical time, and require a trained assistant to be handled.

Postoperative leg length discrepancy (LLD) is a major concern after THA. LLD is regularly associated with patient dissatisfaction and is the major reason for litigation after elective THA worldwide when > 10 mm [21]. Performing THA through the DAA without a table allows the surgeon to examine deliberately the leg length intraoperatively and adapt his steps accordingly. However, there are so far no data in the literature confirming this hypothesis.

Therefore, this match-controlled, retrospective, and single-surgeon study aimed to evaluate and compare the clinical and radiological outcomes, intra- and postoperative complications, and LLD after THA was performed through the DAA with and without the use of a traction table.

## Material and methods

### Patient characteristics

Between January 2015 and December 2018, a total of 1360 THAs were performed in our department. A continuous series of 75 DAA THA (group 1) was performed with the use of a traction table (Schaerer MIS-Extension, Scharer Medical, Münsingen, Switzerland) by a single surgeon (C.T). These cases were matched for age, BMI, and ASA score (Table 1) with 75 patients (group 2) operated without a table by the same surgeon (C.T).

**Table 1:** Patients demographics

	Without table (*N* = 75)	With table (*N* = 75)	Significance (*p* value)
BMI (kg/m^2^)	25 (4)	26 (4.7)	0.63
Age (years)	66 (15.2)	70 (13.4)	0.09
Postoperative Harris Hip Score at 1 year	94 (7.2)	95 (1.5)	0.09
Follow-up (months)	31 (8.6)	43 (7.62)	0.86
ASA Score	2 (0.5)	2.1 (0.6)	0.22
1 (*n*)	7 (9.3%)	5 (6.7%)	
2 (*n*)	48 (64%)	62 (80%)	
3 (*n*)	20 (26.7%)	9 (12%)	
4 (*n*)	0 (0%)	1 (1.3%)	

Values were given as mean and standard deviation

*ASA* American Society of Anesthesiologists, *BMI* body mass index recorded in any group

The inclusion criteria were adult patients undergoing primary THA for symptomatic unilateral hip osteoarthritis through DAA by a single surgeon (C.T) at our institution. Baseline demographics including preoperative Harris Hip Score (HHS), comorbidities likely to influence on postoperative complication rates, and the patient’s physical status according to the American Society of Anesthesiologists (ASA) were recorded. A total of 150 THA through DAA (male, 62; female, 88) with an average age of 68 years (range 29–93) were identified. The average follow-up period was 33 months (range 15–48). This study was performed in line with the principles of the Declaration of Helsinki and approved by our institutional review board (ID 2018-02131).

### Preoperative imaging and templating

All the patients underwent routine preoperative standing anteroposterior pelvic radiograph and anteroposterior and lateral radiographs of the operated hip. In both groups, preoperative templating was performed using the Traumacad software (Traumacad, PetachTikva, Israel) with regard to the femoral osteotomy level, implant size, and positioning, and leg length correction was obtained. The goal of preoperative templating was to restore the native center of rotation of the hip and a similar femoral offset to the sound contralateral side. Any pre-existing leg length discrepancy was corrected as well during templating.

### Surgical technique and perioperative care

All THAs were performed in the supine position. Intraoperative fluoroscopy imaging was used during the insertion of the acetabular component to assess its positioning. A single fellowship-trained hip surgeon (C.T) with adequate experience in minimally invasive anterior approach (> 100 THAs using DAA) performed all the procedures. The implants used in the current cohort were as follows: (1) for the patients under 70 years, a ceramic-on-ceramic (April®, SPS® or Harmony®, Symbios, Yverdon, Switzerland), and (2) for the patients over 70 years, a dual mobility cup construct (Symbol® dual mobility construct, Dedienne Santé, Mauguio, France). Most of the implants used were cementless. The use of cemented Harmony® stems was only necessary in 25 cases as the standard regime in our department is the use of uncemented stems independent of the age of the patient. Bone quality in these 25 patients was considered very poor intraoperatively. Patients which required custom-made implants were not included in the study.

**Fig. 1:**
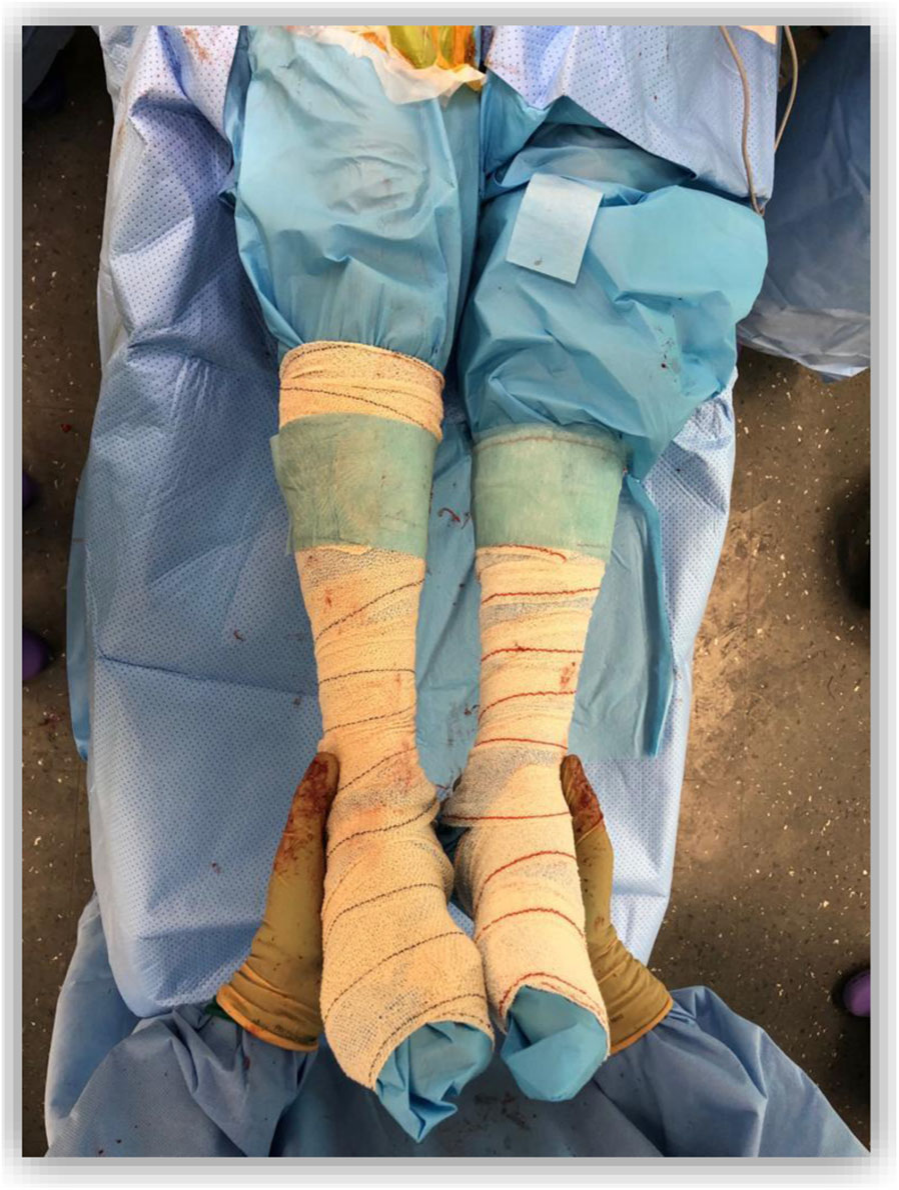
Performing THA through DAA on a normal table

The surgical time was measured from skin incision to wound dressing. When performing THA through the DAA without a table, leg length was examined intraoperatively with the surgeon palpating and comparing both malleoli (Fig. 1). The total operation time was measured from the end of the anesthetic procedure to the transfer to its bed. The blood loss was calculated at the end of the surgery by measuring the fluid accumulation in the suction device after subtracting irrigation and the visual estimation of the blood absorbed by surgical gauze.

All the patients were mobilized out of bed on surgery day with full weight-bearing. A standardized physical therapy protocol was followed. Patients were discharged once they were able to safely perform daily activities, walk on stairs, and once the pain was adequately controlled with oral medications. The hospital stay duration was considered from the day of surgery to hospital discharge.

### Clinical and radiologic evaluation

Patients were followed up clinically and radiographically at 6 weeks, 3 months, 1 year, and then every year. Medical records including outpatient clinic notes, operative reports, hospital records for readmission, and Harris Hip Score were reviewed. The clinical examination was performed in a standardized manner by an orthopedic surgeon not involved in the care or management of the included patients. In the present study, complications considered were excessive intraoperative blood loss requiring transfusion, femoral fracture, dislocation, wound dehiscence and periprosthetic infection.

The postoperative X-rays were evaluated by a junior and a senior orthopedic resident (D.W and G.L) not involved in the patient management and blinded to the patient’s clinical details. The residents performed the measurements individually and concurred with the results. Measurements were performed twice, and the results were evaluated using a single-measure intraclass correlation coefficient (ICC) with a 2-way random effects model for absolute agreement.

A standardized protocol has been applied for obtaining pre- and postoperative radiographs. The lower limbs were held together in a neutral position with the anterior superior iliac spine parallel to each other and to the X-ray table. On the anteroposterior view of the pelvis, the inferior margin of the acetabular teardrop, the most prominent point of the lesser trochanter, and the center of rotation of the femoral head were chosen as reference points. Distances between them were measured to the nearest millimeter. This method does not take into account other discrepancies of length in the lower limb but does give an accurate assessment of the situation before and after surgery [22]. A positive leg length discrepancy value was documented when the operated limb was longer than the contralateral side, and a negative one when it was shorter. Acetabular cup inclination was defined as the angle between the plane of the cup’s bigger diameter and a line bisecting both the acetabular teardrops. Cup anteversion was defined as the angle between the transverse axial plane and the plane of the cup’s bigger diameter in the lateral view [23]. The horizontal and vertical center of rotation (CoR) was defined as the preoperative distance of the CoR to the distance of the postoperative CoR on the horizontal and vertical axes, respectively [24]. A negative value indicated that the postoperative CoR was reconstructed more medial and more superior, respectively.

### Statistical analysis

The achieved power of the study according to the post hoc power analysis with a total sample size of 150 hips, medium effect size, and alpha = 0.05 was 94%. Descriptive statistics are presented as mean ± standard deviation (SD). All parameters were tested for normality to compare normal variables; parametric unpaired *t* tests were used. Otherwise, the Wilcoxon signed-rank tests were used. For the radiological measurements of the cup inclination and anteversion, and leg length, intra- and inter-observer variabilities of the measurements were evaluated by two independent and blinded observers using single-measure intraclass correlation coefficients (ICC) with a 2-way random effects model for absolute agreement. Intra- and inter-observer ICC were respectively 0.93 and 0.91 for cup anteversion, 0.95 and 0.92 for cup inclination, and 0.96 and 0.94 for leg length. The level of statistical significance was set at *p* ≤ 0.05.

## Results

### Functional outcomes, complication rate, and revision

*Intraoperative blood loss was significantly less without a table 520 mL (± 272) than with a traction table (746 mL ± 538), p ≤ 0.05*. There was no significant difference detected in terms of operative time (*p* = 0.13) between both groups. Length of hospital stay was significantly shorter for patients operated without a table (*p* ≤ 0.05) (Table 2). The mean Harris Hip Score (HHS) increased from 58 and 61 preoperatively to 94 (± 7.2) and 95 (± 1.5) at the 1-year follow-up in the traction table group and non-table group, respectively. No significant difference was detected (*p* = 0.09). Regarding complications and revisions, the total reoperation rates in patients operated with the traction table were 4% (three hips) but none without a table (*p* = 0.08). The total complication rates in patients operated with the traction table were 6.7%, compared to 2.6% in the other group (*p* = 0.23). Neither was statistically significant. *There was neither hip dislocation nor femoral fracture in either group*. Causes for complication were non-specific, as reoperations were hematoma needing revision [1], wound healing disorder [1], and infection [1] in the group with a traction table.

**Table 2:** Perioperative parameters

	Without table (*n* = 75)	With table (*n* = 75)	Significance (*p* value)
Intraoperative blood loss (mL)	520 (272)	746 (538)	*p* ≤ 0.05
Surgical time (min)	133 (30)	142 (37)	0.13
Total operation time (min)	198 (38)	199 (38)	0.81
Hospital stay (days)	5.3	6.4	*p* ≤ 0.05

Values were given as mean and standard deviation

### Radiographic findings

*LLD was significantly more accurate in the group without a table (2.4 mm (± 2)) than with a table (3.67 mm (± 3.1)), p ≤ 0.05*. Eight patients (10%) operated without a table lied outside the > 5-mm limit, compared to 15 patients (20%) operated with a traction table. While two patients operated with a traction table were more than 10 mm longer (2.66%), no patient in the other group was within that range (Table 3).

**Table 3:** Radiologic measurements

	Without table (*n* = 75)	With table (*n* = 75)	Significance (*p* value)
Acetabular inclination (°)	41.83 (4.8)	43.68 (4.3)	0.11
Within the “safe zone”	73 (97.3%)	70 (93.3%)	
Acetabular anteversion (°)	29.56 (6)	32 (7)	0.41
Within the “safe zone”	14 (18.6%)	14 (18.6%)	
Vertical center of rotation (mm)	-1.80 (4.4)	-2.48 (5)	0.38
Horizontal center of rotation (mm)	-1.5 (3.9)	-2.3 mm (4.5)	0.36
**Leg length discrepancy** (mm)	2.4 (2)	3.67 (3.1)	*p* ≤ 0.05
- Outliers > 5 mm	8 (10%)	15 (20%)	
- Outliers > 10 mm	0 (0%)	2 (2.66%)	

Values were given as mean and standard deviation

“Safe zone” for acetabular inclination and anteversion was set at 30–50° and 5–25°, respectively, as described by Lewinnek et al. [25]. A positive leg length discrepancy value was used when the operated leg was longer than the contralateral side

No significant difference was detected in the mean acetabular inclination which was similar in both groups (43.4° ± 4.2 in table group, 42° ± 4.8 without a table, *p* = 0.11). No significant difference between both groups was observed neither in the acetabular anteversion (32° ± 7 with a table, 29.56° ± 6 without a table, *p* = 0.41) nor in the modifications of CoR: vertical (− 2.48 mm ± 5 in the table group, − 1.8 mm ± 4.4 without a table, *p* = 0.38) or horizontal (− 2.3 mm ± 4.5 in the table group, − 1.5 mm ± 3.9 without a table, *p* = 0.38).

## Discussion

THA through the direct DAA is a well-established procedure, with low dislocation risk and excellent functional outcomes [25, 26]. Although DAA can be performed with or without the use of a traction table depending on the surgeon’s preference, the majority of the surgeons in Switzerland prefer to use a traction table [27]. To our knowledge, the present study is the only single-surgeon matched-control cohort to evaluate and compare the clinical and radiological outcomes, complications rate, and leg length restoration after THA through DAA with and without the use of a traction table.

No significant difference was detected in terms of acetabular inclination, acetabular anteversion, and center of rotation. The acetabular inclination was identical in both groups, with 95.3% falling in the “safe zone” as described by Lewinnek et al. [28]. Moreover, acetabular anteversion showed no significant difference between both groups. Regarding the vertical and horizontal CoR, no significant difference was observed between both groups. In the group without a traction table, a slightly more accurate reconstruction of leg length was achieved with a mean of 2.14 mm (range 0–8.6 mm), compared to the group with a traction table, with a mean of 3.67 mm (range 0–14.2 mm).

**Fig. 2:**
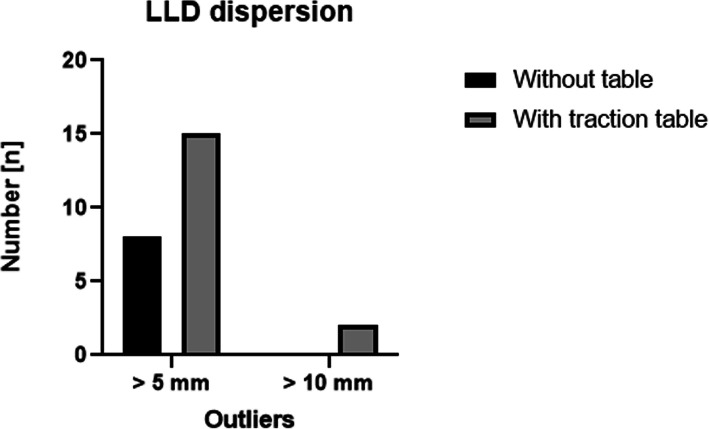
LLD dispersion: number of outliers in the 5- and 10-mm ranges in both groups

The most important finding of this present study is that *performing THA through DAA in a standard table might be helpful for better reproducibility in restoring leg length (p* ≤ *0.05)*. Only two patients operated with the traction table presented a LLD > 10 mm (2.6%). There was none in the standard table group (Fig. 2). Some studies with a traction table previously reported a mean LLD between 1 and 7 mm [15, 29–31], with 11% showing an LLD > 10 mm while Batailler et al. [32] performing THA through DAA without a traction table showed a mean LLD of 2 mm, which is comparable to our results. Any observed radiographic differences are likely subclinical as no difference in clinical outcome was observed.

Perfect restoration of leg length is undoubtedly desired after THA as LLD has been associated with back pain and sciatica, neuritis, gait disorder, and general dissatisfaction [33–35]. However, LLD is not uncommon after THA. In the literature, LLD varies [36] with a mean from 3 to 17 mm [37, 38]. Between 6% [39] and 32% [40] of patients noted their LLD. As Desai et al. reviewed [21], Love and Wright [39] reported up to 18% of patients had lengthening of more than 1.5 cm, of whom 6% required shoe correction. Although the boundaries between acceptable and unacceptable levels of LLD remain undefined [41], it is universally perceived pathological when shortening exceeds 10 mm and lengthening 6 mm [40].

**Fig. 3:**
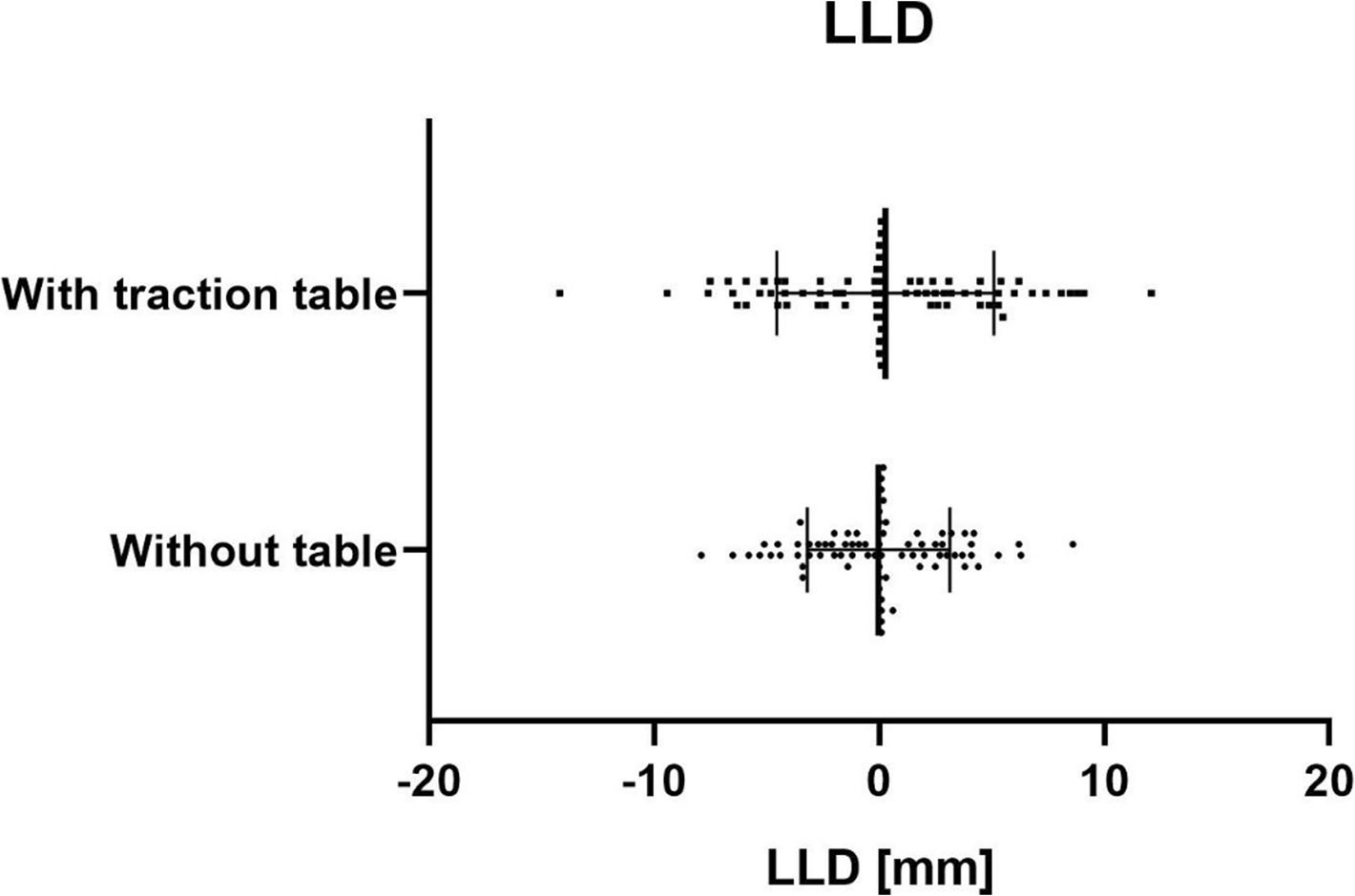
Scatter diagram of LLD (mm) in both groups

However, some authors [40] conclude that even a small disparity in leg length may be a source of dissatisfaction for patients. Furthermore, some authors conclude that patients can detect a relatively minor increase in leg length and are unsatisfied with the use of compensatory inshoes [42]. By setting the cutoff for LLD at 5 mm which could be considered quite a strict value, compared to the universally accepted values, 15 patients (20%) were lengthened more than 5 mm in the cohort of patients operated with a table. All were longer. In the group operated without a traction table, only 10 (13.3%) patients presented an LLD greater than 5 mm (Fig. 3). Undoubtedly, it is debatable if these differences have any impact on the clinical outcome particularly if we consider that there was no significant difference in the HHS after 1 year. However, it is questionable if the HHS is a sufficient tool in detecting the impact of minor discrepancies in leg length. *While THA through DAA is a demanding technique, with a steep learning curve, less outliers without a traction table might indicate a better reproducibility and thus help surgeons to reduce their outliers when performing THA through DAA*.

Furthermore, our results showed significantly less blood loss when performing THA through DAA in a standard table (*p* ≤ 0.05), but no significant difference in surgical time and functional outcome (Table 2). In our opinion, it is not evident why blood loss was higher in the table cohort. It could be explained by the slightly elevated duration of surgery in this group (+ 9 min), even though not significant (*p* = 0.13). Another hypothesis could be that in the table group, repetitive maneuvers might have been necessary to expose the femur, resulting in a slightly higher blood loss. The average length of stay in the hospital was significantly shorter for patients operated without a table (*p* ≤ 0.05). Although THA through DAA has already been shown to be related to a lower hospital stay compared to THA through a posterior approach [4], there is still no evidence in the literature that THA through DAA without a table might be more cost-effective when compared to THA through DAA with a traction table. Other studies might be conducted to compare the efficiency and cost-effectiveness of each technique.

There was no significant difference between both groups regarding complication and reoperation rates. No dislocation occurred in either group, which confirms the low rates reported in the literature (0.96–1.5%) [43–45]. Perforation of the femoral canal and trochanteric fractures has been associated with the DAA [19, 44]. Moreover, the femoral shaft and ankle fracture have been contributed directly to the use of a traction table [15]. None of these specific complications was found in our study. We had a 6.7% total complication rate in the group operated with a traction table, compared to a 2.6% total complication rate in the other standard table group.

This study should be interpreted in light of its potential limitations. The main limitation of this study is the retrospective design. However, due to the good documentation in our department, most of the patients’ data required for the current study were available. Moreover, radiological analyses were performed using plain radiographs instead of CT scan or EOS imaging, which might have increased the precision in assessing the radiological outcomes. Additionally, the changes in leg length were minor and had no impact on the clinical outcome. However, the single-surgeon cohort adds to the strength of our study.

## Conclusion

In conclusion, this study is to our knowledge the first single-surgeon matched-control study available in the literature comparing THAs through DAA with and without a traction table. The results of our study suggest that performing THA through the DAA without a traction table might lead to more reliable results concerning LLD (LLD is more accurate in the group without a table, *p* ≤ 0.05) and reduce significantly the outliers (> 10 mm difference), when compared to performing THA through the DAA with a traction table. There was no difference in peri- and postoperative complications or component positioning. The growing interest for less invasive arthroplasty and the anterior approach makes this result interesting. However, further larger studies may be needed to confirm these results.

## Data Availability

The datasets used and analyzed during the study are available from the corresponding author on reasonable request.

## References

[1] Petis S, Howard JL, Lanting BL, Vasarhelyi EM (2015). Surgical approach in primary total hip arthroplasty: anatomy, technique and clinical outcomes. Can J Surg.

[2] York PJ, Smarck CT, Judet T, Mauffrey C (2016). Total hip arthroplasty via the anterior approach: tips and tricks for primary and revision surgery. Int Orthop..

[3] Chechik O, Khashan M, Lador R, Salai M, Amar E (2013). Surgical approach and prosthesis fixation in hip arthroplasty world wide. Arch Orthop Trauma Surg..

[4] Barrett WP, Turner SE, Leopold JP (2013). Prospective randomized study of direct anterior vs postero-lateral approach for total hip arthroplasty. J Arthroplasty..

[5] Lecoanet P, Vargas M, Pallaro J, Thelen T, Ribes C, Fabre T. Leg length discrepancy after total hip arthroplasty: can leg length be satisfactorily controlled via anterior approach without a traction table? Evaluation in 56 patients with EOS 3D. Orthop Traumatol Surg Res. 2018;104(8):1143–48. 10.1016/j.otsr.2018.06.020 30314938

[6] Dimitriou D, Helmy N, Hasler J, Flury A, Finsterwald M, Antoniadis A (2019). The role of total hip arthroplasty through the direct anterior approach in femoral neck fracture and factors affecting the outcome. J Arthroplasty.

[7] Swiss National Joint Registry, SIRIS Report 2012-2016.

[8] Antoniadis A, Dimitriou D, Flury A, Wiedmer G, Hasler J, Helmy N (2018). Is direct anterior approach a credible option for severely obese patients undergoing total hip arthroplasty? A matched-control, retrospective, clinical study. J Arthroplasty.

[9] Taunton MJ, Trousdale RT, Sierra RJ, Kaufman K, Pagnano MW (2018). John Charnley Award: randomized clinical trial of direct anterior and miniposterior approach THA: which provides better functional recovery?. Clin Orthop.

[10] Wang Z, Hou J, Wu C, Zhou Y, Gu X, Wang H, et al. A systematic review and meta-analysis of direct anterior approach versus posterior approach in total hip arthroplasty. J Orthop Surg. 2018;13(1) https://josr-online.biomedcentral.com/articles/10.1186/s13018-018-0929-4. [cité 25 oct 2018]. 10.1186/s13018-018-0929-4 PMC6127950 30189881

[11] Jia F, Guo B, Xu F, Hou Y, Tang X, Huang L (2019). A comparison of clinical, radiographic and surgical outcomes of total hip arthroplasty between direct anterior and posterior approaches: a systematic review and meta-analysis. Hip Int J Clin Exp Res Hip Pathol Ther.

[12] Hueter C. Funfte abtheilung: die verletzung und krankheiten des huftgelenkes neunundzwnzigtes capitel. In: Hueter C (ed) Grundriss der chirurgie, 1883, 2nd edn. Leipzig: FCW Vogel; pp 129–200.

[13] Rachbauer F, Kain MSH, Leunig M (2009). The history of the anterior approach to the hip. Orthop Clin North Am.

[14] Judet J, Judet H (1985). Anterior approach in total hip arthroplasty. Presse Medicale Paris Fr 1983.

[15] Matta JM, Shahrdar C, Ferguson T (2005). Single-incision anterior approach for total hip arthroplasty on an orthopaedic table. Clin Orthop.

[16] Matta JM, Ferguson TA (2005). The anterior approach for hip replacement. Orthopedics..

[17] Manrique J, Chen AF, Heller S, Hozack WJ. Direct anterior approach for revision total hip arthroplasty. Ann Transl Med. 2014;2(10) https://www.ncbi.nlm.nih.gov/pmc/articles/PMC4205864/. [cité 29 nov 2019]. 10.3978/j.issn.2305-5839.2014.09.11 PMC4205864 25405154

[18] De Geest T, Fennema P, Lenaerts G, De Loore G (2015). Adverse effects associated with the direct anterior approach for total hip arthroplasty: a Bayesian meta-analysis. Arch Orthop Trauma Surg.

[19] Jewett BA, Collis DK (2011). High complication rate with anterior total hip arthroplasties on a fracture table. Clin Orthop Relat Res.

[20] Flierl MA, Stahel PF, Hak DJ, Morgan SJ, Smith WR (2010). Traction table-related complications in orthopaedic surgery. J Am Acad Orthop Surg..

[21] Desai AS, Dramis A, Board TN (2013). Leg length discrepancy after total hip arthroplasty: a review of literature. Curr Rev Musculoskelet Med.

[22] Sayed-Noor AS, Hugo A, Sjödén GO, Wretenberg P (2009). Leg length discrepancy in total hip arthroplasty: comparison of two methods of measurement. Int Orthop..

[23] Woo RY, Morrey BF (1982). Dislocations after total hip arthroplasty. J Bone Joint Surg Am.

[24] Fessy MH, N’Diaye A, Carret JP, Fischer LP (1999). Locating the center of rotation of the hip. Surg Radiol Anat.

[25] Restrepo C, Parvizi J, Pour AE, Hozack WJ (2010). Prospective randomized study of two surgical approaches for total hip arthroplasty. J Arthroplasty.

[26] Meermans G, Konan S, Das R, Volpin A, Haddad FS (2017). The direct anterior approach in total hip arthroplasty: a systematic review of the literature. Bone Jt J.

[27] (11) (PDF) SIRIS Report 2019. Annual Report of the Swiss National Joint Registry, Hip and Knee, 2012 – 2018 [Internet]. ResearchGate. [cité 20 mars 2020]. Disponible sur: https://www.researchgate.net/publication/337533256_SIRIS_Report_2019_Annual_Report_of_the_Swiss_National_Joint_Registry_Hip_and_Knee_2012_-_2018.

[28] Lewinnek GE, Lewis JL, Tarr R, Compere CL, Zimmerman JR (1978). Dislocations after total hip-replacement arthroplasties. J Bone Joint Surg Am.

[29] Woolson ST, Pouliot MA, Huddleston JI (2009). Primary total hip arthroplasty using an anterior approach and a fracture table: short-term results from a community hospital. J Arthroplasty.

[30] Müller DA, Zingg PO, Dora C (2014). Anterior minimally invasive approach for total hip replacement: five-year survivorship and learning curve. Hip Int J Clin Exp Res Hip Pathol Ther.

[31] Bhandari M, Matta JM, Dodgin D, Clark C, Kregor P, Anterior Total Hip Arthroplasty Collaborative Investigators (2009). Outcomes following the single-incision anterior approach to total hip arthroplasty: a multicenter observational study. Orthop Clin North Am.

[32] Batailler C, Fary C, Batailler P, Servien E, Neyret P, Lustig S (2017). Total hip arthroplasty using direct anterior approach and dual mobility cup: safe and efficient strategy against post-operative dislocation. Int Orthop.

[33] Mihalko W, Phillips M, Krackow K (2001). Acute sciatic and femoral neuritis following total hip arthroplasty: a case report. J Bone Jt Surg-Am.

[34] Friberg O (1983). Clinical symptoms and biomechanics of lumbar spine and hip joint in leg length inequality. Spine..

[35] Rösler J, Perka C (2000). The effect of anatomical positional relationships on kinetic parameters after total hip replacement. Int Orthop..

[36] Sathappan SS, Ginat D, Patel V, Walsh M, Jaffe WL, Di Cesare PE (2008). Effect of anesthesia type on limb length discrepancy after total hip arthroplasty. J Arthroplasty.

[37] Turula KB, Friberg O, Lindholm TS, Tallroth K, Vankka E. Leg length inequality after total hip arthroplasty. Clin Orthop Relat Res. 1986;(202):163–8. 3955944

[38] Rand JA, Ilstrup DM. Comparison of Charnley and T-28 total hip arthroplasty. Clin Orthop. 1983;(180):201-5. 6627789

[39] Love B, Wright K (1983). Leg-length discrepancy after total hip replacement. J Bone Jt Surg Am..

[40] Edeen J, Sharkey PF, Alexander AH (1995). Clinical significance of leg-length inequality after total hip arthroplasty. Am J Orthop Belle Mead NJ.

[41] Dougall TW, White TO (2002). Arthroplasty of the hip. Leg length is not important. J Bone Joint Surg Br.

[42] Maloney WJ, Keeney JA (2004). Leg length discrepancy after total hip arthroplasty. J Arthroplasty.

[43] Lee G-C, Marconi D (2015). Complications following direct anterior hip procedures: costs to both patients and surgeons. J Arthroplasty..

[44] Sariali E, Leonard P, Mamoudy P (2008). Dislocation after ttal hip arthroplasty using Hueter anterior approach. J Arthroplasty.

[45] Siguier T, Siguier M, Brumpt B (2004). Mini-incision anterior approach does not increase dislocation rate: a study of 1037 total hip replacements. Clin Orthop..

